# Reconsideration of Plant Morphological Traits: From a Structure-Based Perspective to a Function-Based Evolutionary Perspective

**DOI:** 10.3389/fpls.2017.00345

**Published:** 2017-03-15

**Authors:** Shu-Nong Bai

**Affiliations:** State Key Laboratory of Protein and Plant Gene Research, College of Life Science, Quantitative Biology Center, Peking UniversityBeijing, China

**Keywords:** morphological traits, function-based evolutionary perspective, sexual reproduction cycle, plant developmental unit, plant morphogenesis 123

## Abstract

This opinion article proposes a novel alignment of traits in plant morphogenesis from a function-based evolutionary perspective. As a member species of the ecosystem on Earth, we human beings view our neighbor organisms from our own sensing system. We tend to distinguish forms and structures (i.e., “morphological traits”) mainly through vision. Traditionally, a plant was considered to be consisted of three parts, i.e., the shoot, the leaves, and the root. Based on such a “structure-based perspective,” evolutionary analyses or comparisons across species were made on particular parts or their derived structures. So far no conceptual framework has been established to incorporate the morphological traits of all three land plant phyta, i.e., bryophyta, pteridophyta and spermatophyta, for evolutionary developmental analysis. Using the tenets of the recently proposed concept of sexual reproduction cycle, the major morphological traits of land plants can be aligned into five categories from a function-based evolutionary perspective. From this perspective, and the resulting alignment, a new conceptual framework emerges, called “Plant Morphogenesis 123.” This framework views a plant as a colony of integrated plant developmental units that are each produced via one life cycle. This view provided an alternative perspective for evolutionary developmental investigation in plants.

## Introduction

According to the Oxford Dictionary, a “trait” is defined as “a distinguishing quality or characteristic, typically one belonging to a person” and more specifically, a “genetically determined characteristic.” Biologists know that characteristics ranging from a single base pair of nucleic acid to the overall shape of an organism can be determined genetically. Therefore, traits are meaningful only when considered in a specific context. Here, I will discuss traits in terms of morphology and consider morphological traits from a function-based evolutionary perspective.

Where and how present-day organisms were originated in the biological world are long-lasting, fundamental questions. While Darwin’s theory of evolution established a conceptual framework, the survival of fittest under natural selection, for answering such questions, the detailed mechanisms remain elusive. For many years, the ancestral relationships or family lineages among species, i.e., phylogeny, were mainly determined based on the similarity of morphological traits. During the past decades, one of the most impressive advances in biology was the discovery, based on mutant analyses, that many complicated morphological traits are determined by a single or a few proteins encoded by genes inherited in a Mendelian manner and that some such genes are conserved across species. This discovery prompted scientists to explore whether innovations in morphological traits during evolution were associated with a gain or loss of the genes determining these traits. Such analyses launched the field of “evolutionary developmental biology,” abbreviated as “Evo-Devo” (Gilbert et al., 1996; Goodman and Coughlin, 2000; Raff, 2000; Gilbert, 2003).

Evo-Devo studies initially focused on comparisons of animal genes. It has long been known that most animal individuals develop from a single cell (zygote) and that their morphological traits emerge or form primarily through embryogenesis. This common morphogenetic pattern, centering mainly on embryogenesis, establishes a framework for comparing morphological traits among different species.

Investigations of plants, like those of animals, began with observations of their appearance, or “surface” according to Gifford and Foster (1989). Traditionally, a plant was considered to be consisted of three parts, i.e., the shoot, the leaves, and the root (Strasburger et al., 1976). Based on such a “structure-based perspective,” evolutionary analyses or comparisons across species were made on particular parts or their derived structures. However, morphogenetic patterns in plants are fundamentally different from those of animals. One of the most prominent differences is that no visible process equivalent to animal embryogenesis has been observed in the plant kingdom (Waddington, 1966). Some authors have defined plant embryogenesis as encompassing the period from zygote to seed formation (Goldberg et al., 1994). If this definition is valid, mosses and ferns do not undergo “embryogenesis,” since these plants do not produce seeds. The concept of “alternation of generations” was proposed by Hofmeister in the 1850s (Kaplan and Cooke, 1996). This concept, i.e., that all the land plants have both sporophyte (diploid) and gametophyte (haploid), has been used as a framework for comparing morphological traits among diverse species. In recent years, much effort has been devoted to explore evolutionary innovations focusing on the particular parts or their derived structures across phyla (reviewed by Harrison, 2017). However, most of these studies have involved comparing closely related angiosperm species (Irish and Benfey, 2004; Preston et al., 2011; Della Pina et al., 2014). This situation aroused an enthusiastic discussion at a recent NPH symposium about whether there are unique themes that define plant Evo-Devo (Liao et al., 2016).

It is perfectly legitimate to choose any morphological trait, such as the number or color of spots on a petal or the formation of root hairs, as a target for exploring the underlying regulatory mechanism and its origin in closely related species (Martins et al., 2016) or even across phyla (Menand et al., 2007). These efforts underscore the well-established principle that new traits emerge from interactions between genetic variation and environmental selection, with some details differing among studies. However, core questions in evolutionary theory related to plants remain, such as how photoautotrophic organisms diverged from a common ancestor, and what key evolutionary innovations resulted in the divergence of the major lineages.

Advances in DNA sequencing technology make it no longer difficult to obtain genome information for a species of interest, nor to find differences among genomes used for comparison. The problem is how to determine what these sequence differences truly mean. Two questions need to be answered: whether the differences in DNA sequences are responsible for particular traits, and whether the traits in the species being compared are evolutionarily related. It is relatively easy to determine the causal relationship between a single DNA sequence and the targeted trait through modern genetic analysis. However, similarity of DNA sequences may not necessarily indicate evolutionary relationships of traits of interest, as protein complexes, metabolic processes, and regulatory networks (in short, cellular functions) are highly complex and approximately 10 times more proteins than genes have been annotated. From this perspective, elaborating evolutionary innovations or relationships between traits is beyond the scope of DNA sequence analysis.

Traditionally, morphology deals with the study of the form and structure of an organism. Recent Evo-Devo studies have explored the relationships between morphological traits and the (possibly) corresponding genes from an evolutionary perspective. However, a fundamental element has largely been neglected in plant Evo-Devo studies, the role of photoautotrophism. If we are asked to identify the most basic difference between plants and animals, the best answer is likely their manner of energy acquisition: plants are photoautotrophic and animals are heterotrophic. Considering the essential roles of the efficiency of energy acquisition and environmental adaptation in the evolutionary selection of morphological traits, if we analyze morphological traits from a function-based evolutionary perspective rather than structure-based perspective, derived from the tradition of morphology, could we uncover a new scenario?

## Underlying Principles for Investigations Based on A Function-Based Evolutionary Perspective

Based on the current literature, morphological traits can be grouped in roughly three classes: one includes morphological traits investigated due to personal interest, such as sepal color or spots on petals; another comprises those with application significance, such as crop productivity and quality; and the third includes those with evolutionary importance, such as vascular tissues, seeds and flowers, associated with particular taxonomic groups. These ways of grouping and comparisons of morphological traits are all derived from the traditional structure-based perspective. To align morphological traits from a function-based evolutionary perspective, some background information is needed.

**FIGURE 1:**
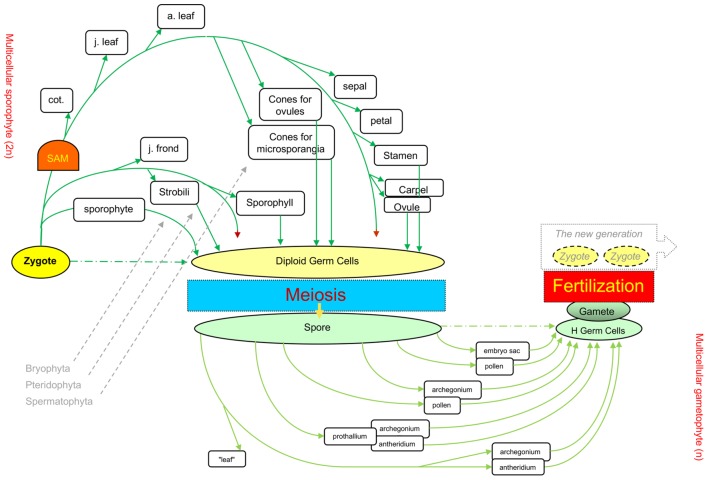
**Different levels of elaboration around the core processes in the life cycles of the three plant phyla.** The sexual reproduction cycle (Bai, 2015a) from one zygote to the next generation’s zygotes through meiosis and fertilization is the backbone of the lifecycle for all three land plant groups, Bryophyta, Pteridophyta, and Spermatophyta. Green arrows show the differentiation of various organ types in diploid phase, and light green for organs in haploid phase. Dark red arrowheads indicate unlimited tip growth activity. cot., cotyledons; j. leaf, juvenile leaf (e.g., rosette leaves in *Arabidopsis*); a. leaf, adult leaf (e.g., cauline leaves in *Arabidopsis*). This figure was revised from Figure 1.9 in Bai and Xu (2013).

The current mainstream concept of plant developmental programs (the underlying mechanism for morphogenesis) states that plants have an indeterminate developmental program (Goldberg, 1988). However, when all land plants are considered, their life cycles include clear starting and ending points, i.e., zygotes and gametes, respectively. Between these two points, another unique cell turns from diploid to haploid, i.e., the meiotic cell (which arises from the diploid germ cell, DGC), leading to meiosis and spore formation (Bai, 1999, 2015a; Bai and Xu, 2013; Zhao et al., 2017; **Figure 1**).

The core process of the eukaryotes life cycle comprises three unique or core cells (zygotes, meiotic cells, and gametes), which serve as reference points, and three events that occur at the unicellular level, i.e., meiosis, fertilization, and heterogametogenesis (Bai, 1999). Such a core process was recently described as an ancestral process originating from unicellular eukaryotes, and designated as the sexual reproduction cycle (SRC) (Bai and Xu, 2013; Bai, 2015a). The SRC represents a modified cell cycle that functions as the ultimate mechanism that helps eukaryotes adapt to unpredictable environmental changes and serves as a backbone upon which multicellular organisms are derived via the interpolation of multicellular structures into the two (diploid and haploid) intervals of the life cycle (Bai, 2015a; **Figure 1**).

**FIGURE 2:**
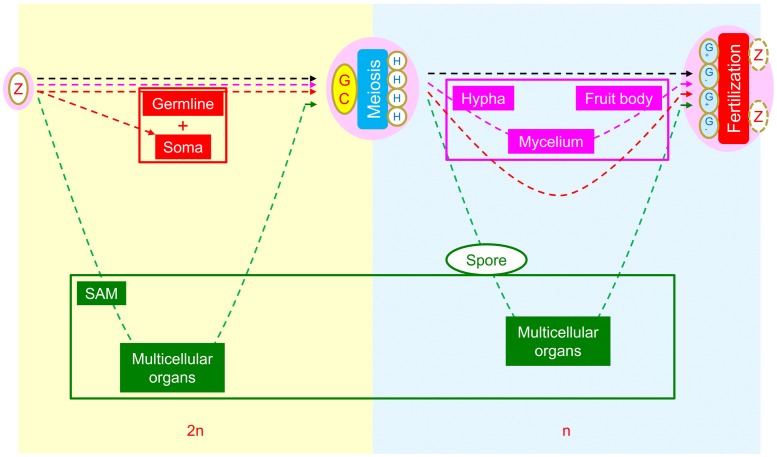
**Comparison of morphogenetic strategies of animals, fungi, and plants within the framework of the SRC.** Yellow background indicates the diploid phase and blue background indicates the haploid phase. In the intervals between zygote and diploid germ cells, the interpolation of multicellular structures occurs in animals (red) and plants (green), whereas none are present in fungi (pink). In the intervals between meiotically produced cells and gametogenic cells, the interpolation of multicellular structures occurs in fungi and plants but not in animals. Reprinted from Bai (2015a).

**FIGURE 3:**
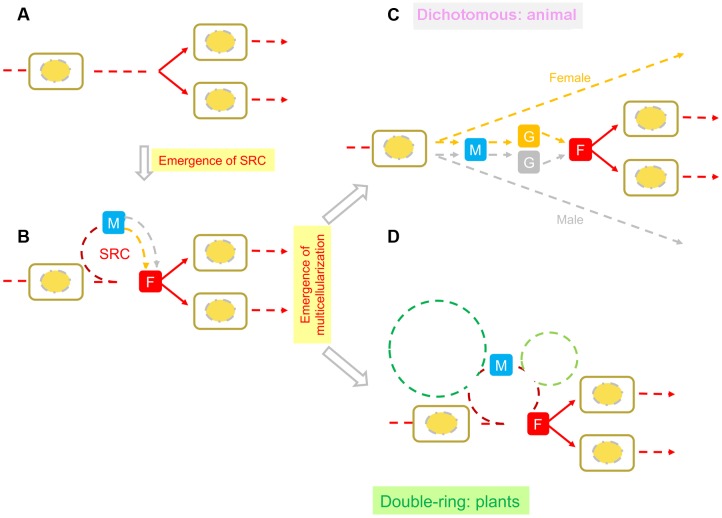
**Comparison of SRC-derived developmental modes in animals and plants.** **(A)** Represents a cell cycle, i.e., one diploid cell becomes two cells through mitosis; **(B)** Represents the SRC (sexual reproduction cycle, Bai, 2015a). Two arrows (orange and gray) between M (meiosis) and F (fertilization) represent heterogametogenesis. **(C)** Represents the “dichotomous mode” for animal development. Orange and gray lines represent female and male soma and germlines, respectively, and orange and gray Gs represent female and male gametogenesis, respectively. **(D)** Represents the “double-ring mode” of plant development. On the SRC backbone, the green dashed ring on the left represents diploid multicellular structures composed of various types of organs; the light green ring on the right represents haploid multicellular structures composed of various types of organs. This figure was modified from Figure 16 in Bai (2016).

Possibly owing to their different manners of energy acquisition, i.e., photoautotrophism for plants and heterotrophism for animals and fungi, three different patterns of interpolation of multicellular structures have evolved in animals, fungi, and plants (**Figure 2**). The formation of multicellular structures in the animal and fungal kingdoms is interpolated once into the first (diploid) and second (haploid) intervals of the SRC respectively. By contrast, the formation of multicellular structures in the plant kingdom is interpolated twice: into both intervals of the SRC. The different patterns of interpolation in multicellular structure formation in animals versus plants results in two different developmental programs (Bai, 2015b, 2016; **Figure 3**). Animals develop via the “dichotomous mode,” meaning that cells derived from the zygote soon diverge into two lineages: one that differentiates into the germline, functioning as a carrier of the SRC, and one that differentiates into the soma, functioning in energy acquisition and environmental responses. The early stages of soma differentiation, together with germline development, can be considered to represent embryogenesis. By contrast, plants develop via the “double-ring mode,” meaning that all cells derived from the zygote differentiate into somatic structures required for photoautotrophy. In response to external and internal stress, along with increased photosynthetic tissue area, some cells are induced to differentiate into DGCs to help the plant prepare to adapt to these stresses through autonomous genetic variations generated via meiosis. This process represents the first ring, from zygote to DGCs via the formation of sporophytes with sequentially formed organ types (e.g., in *Arabidopsis*: cotyledons, rosette and cauline leaves, sepals, petals, stamens, and carpels). After meiosis, multicellular structure formation is interpolated into the second interval of the SRC, from spores into haploid germ cells (HGCs, which differentiate into gametes), and the second ring (gametophyte) is formed.

**FIGURE 4:**
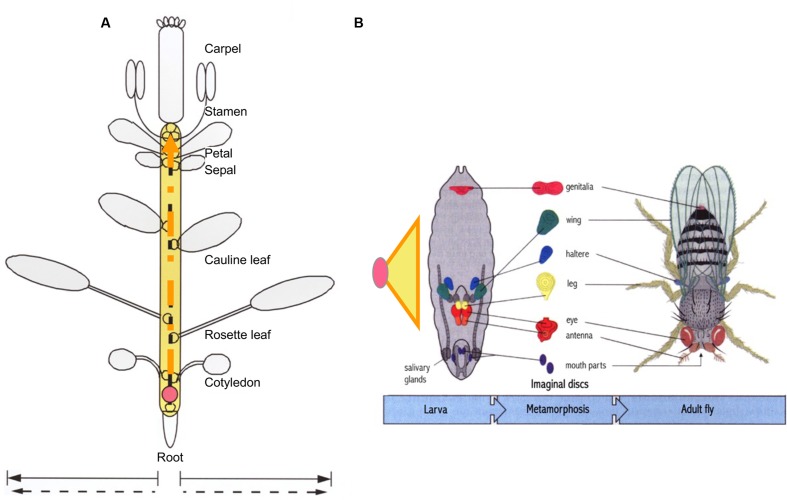
**Comparison of developmental units in plants and animals required for life-cycle completion.** While plant growth tip generated from a zygote (pink circle) can produce numerous lateral organs and branches, only seven organ types in *Arabidopsis* are present in a complete the life cycle **(A)**. The half circles along the dashed orange arrow represent organ primordia. By comparison, the basic structure required for *Drosophila* to complete its life cycle is the embryo, elaborated from a zygote to larva. Embryogenesis is represented by an orange-lined yellow triangle **(B)**. Unlike animal individuals, which contain limited types and numbers of organs in a determined pattern, the functionally equivalent structure in plants is the imaginal unit shown in **(A)**, referred to as a “developmental unit,” rather than the whole plant. According to this perspective, the structural equivalent of an animal embryo would be the process represented as the yellow area in **(A)**. Orange dashed lines in the yellow region indicate that the process is relatively open but ultimately limited. **(B)** Was modified from Figures 2–6 in Wolpert et al. (2007) edited Principles of Development. This figure was modified from Figure 6 in Bai (1999).

Based on the above view, if embryogenesis (consisting of soma and germline development, i.e., dichotomous development) is the core process shared by all animal species during morphogenesis, then double ring development can be considered the core process shared by all plant species during their morphogenesis. Taking *Arabidopsis* as an example, the multicellular structures carrying out the core process in the first (diploid) ring can be thought of as a combination of limited types (not numbers) of organs derived from the growth tip (**Figure 4**). Such a combination has been designated as a “plant developmental unit (PDU)” (Bai, 1999; Bai and Xu, 2013). The early development of these organs (i.e., the primordia) is therefore functionally equivalent to animal embryogenesis, and was designated as a “virtual embryo” by Da-Ming Zhang (Institute of Botany, Chinese Academy of Sciences), in contrast to the physical animal embryo (a “visual embryo”; Bai, 2016).

From this perspective, it becomes clears that we can use the “SRC-derived double ring” as a frame of reference to align morphological traits for Evo-Devo analysis. As morphological traits are regarded as evolutionary consequences of adaptation to improve energy acquisition (photosynthesis), environmental responses (particularly for SRC completion), and growth in extreme environments, I refer to this view of trait description and classification as a “function-based evolutionary perspective.”

## Alignment of Traits from A Function-Based Evolutionary Perspective

Traditionally, plant morphology refers to investigations of “hidden aspects of form, structure, and reproduction that constitute the bases for the interpretation of similarities and differences among plants” (Gifford and Foster, 1989). Although more sophisticated tools, including microscopy, genetics, and molecular biological tools, have progressively been developed and applied to these types of investigations, all of the targeted phenomena, i.e., morphological traits, are initially described based on forms or “surface perspectives” observed by the human eye and interpreted through the faculty of reasoning. It is therefore understandable that observers after the 18th century treated plants as individuals (like animal individuals) comprising three major parts: the shoot, the leaves, and the root (Strasburger et al., 1976). Morphological traits were compared among the structures of “individuals” of various species, primarily following the principles of homology and analogy, referred here as a structure-based perspective. However, the founding fathers of modern botany, such as Grew and Malpighi in the 17th century, treated a plant as a colony, in which each bud is treated as an individual that completes a life cycle (review in Arber, 1950). Although this insightful concept has been marginalized by the mainstream community of modern plant morphologists, it was utilized by a few scholars such as Waddington (1966), who wrote that “a branch… gives rise to a whole new cycle of growth and development.” The concept of a SRC-derived double ring mode of plant development (as described above, with branches representing partial units, as they generally produce organ types that had not yet formed from where the buds had initiated) echoes and is reviving this classic concept (Bai, 1999, 2015a,b, 2016; Bai and Xu, 2013). In the remainder of this article, I will attempt to align morphological traits from a function-based evolutionary perspective.

First, morphological traits must be classified into unicellular and multicellular traits. As mentioned above, SRC first evolved in unicellular eukaryotes. All differentiation processes and interactions completed and exhibited at the unicellular level could be classified as morphological traits at the unicellular level. Included in this class are cell shape, size, and structure; cell division and fusion; and modified cell cycle, i.e., the SRC (consisting of three core cell types and the three core events mentioned above). However, due to space constraints, these traits will not be discussed here.

The other class of morphological traits includes those exhibited at the multicellular level. These traits can be further classified into five categories: (1) those associated with the formation of multicellular structures facilitating photosynthesis and therefore representing outgrowth of a larger structure from the unicellular SRC; (2) those exhibiting diversified differentiation of multicellular structures upon exposure to internal and external stresses, such that structures become smaller and finally return to the unicellularity of the SRC; (3) those derived to ensure heterogametogenesis (sex differentiation); (4) those facilitating the completion of the SRC and the life cycle (sex behavior); and (5) those derived for adaptation to extreme or particular environmental stresses.

### Morphological Traits Facilitating Photosynthesis

**Table 1** shows an alignment of morphological traits that facilitate photosynthesis from the function-based evolutionary perspective. In this category, the key function is facilitating photosynthesis. The multicellular structures required for this function can form in both diploid (sporophytes) and haploid (gametophytes) in all three land-plant phyla, i.e., bryophytes (mainly gametophytes), pteridophytes (both sporophytes and gametophytes), and spermatophytes (mainly sporophytes). The differentiation of multicellular structures can be further grouped into three subcategories: (1) basic structures for maximizing photosynthetic surface area (facilitating energy acquisition), e.g., linear or columnar structures (filaments/twigs/stems), foliage structures (leaves), and branches; (2) multicellularized growth tips; and (3) structures for optimizing photosynthesis, such as stomata.

**Table 1:** Alignment of morphological traits facilitating photosynthesis from a function-based evolutionary perspective.

	Subcategories	Underlying factors				Morphology observed		Reference
Form of multicellular structures for photosynthesis, away from unicellularity in the SRC	Axial growth for increase of photosynthetic surface area	Linear growth	Region	Speed		The combination of growth region and speed results in diversified patterns in axial structures, which serve as basic elements for more complicated structures such as stems, leaves, and roots (a particularly stem-like structure)		Ligrone et al., 2012; Raven and Edwards, 2001; Prusinkiewicz and Lindenmayer, 1990
			Tip	Quick, slow, and stopped				
			Middle					
			Base					
		Branching	Single plane	Asymmetric		Alternate branching of stems		Gifford and Foster, 1989 Tsukaya, 2014; Scagel, 1984
				Combination of symmetric and asymmetric	Non-webbed	Opposite branching of stems		
					Webbed	Foliage structures (leaves) in all three phyla (bryophytes, pteridophytes, and spermatophytes)		
			Multiple planes			Other forms of branching, e.g., spiral		

	Multicellularized growth tip	Randomly arranged				Gymnosperm SAM		Gifford and Foster, 1989
		Layered arranged				Angiosperm SAM		

	Optimizing photosynthesis	CO_2_: opening to air				Stomatal complex		Han and Torii, 2016
		H_2_O		Absorption		Root hairs and rhizoids		Jones and Dolan, 2012
				Transportation		Vascular bundles	Xylem	Lucas et al., 2013
							Phloem	
							Cambium	
		Light: phototropism				Particular cell type or differential growth		Briggs, 2014


From this perspective, it is clear that the morphological traits considered in traditional morphology, such as shoots, leaves, and roots, are derived from combinations of the elements listed in **Table 1**. It also elucidates why the Lindenmayer’s L-system, elaborated by Prusinkiewicz, which treats plant morphogenesis as an “axil tree” following “rewriting” rules, is so powerful for simulating plant morphology and morphogenetic processes (Prusinkiewicz and Lindenmayer, 1990^1^). Such astonishing success implies that there must be simple principles underlying plant morphogenesis, in contrast to the traditional belief that the rules for plant morphogenesis are species-specific and difficult to define. Furthermore, from this perspective, it is clear that, as suggested by the L-system, plant morphogenesis is carried out by the repeated use of similar principles or rules to generate similar structures with modifications, resulting in endless branching. This concept explains the insight the founding fathers proposed: that each bud as an individual to complete its life cycle. The next challenge is to identify the molecular mechanisms underlying these “simple principles.”

### Morphological Traits Associated with Decrease of Multicellularity upon Exposure to Stress

As mentioned above, SRC was proposed to represent the ultimate mechanism that allows plants adapt to unpredictable environmental changes (Bai, 2015a). This mechanism facilitates adaptation by autonomously increasing genetic variations through meiosis and transmitting the best adaptations to the next generation through fertilization. Since plants are photoautotrophic organisms, they acquire energy through photosynthesis: the larger the surface area available for photosynthesis, the better. However, the larger the photosynthetic surface, the larger its interface with the environment, increasing the requirement for the plant to cope with unpredicted environmental changes and for internal mechanical support for this large surface. This internal mechanical support, as far as we know, comes from the cell wall. In turn, cell walls generate internal mechanic stress. This factor, along with the increase in photosynthetic surface area, increases internal/external stresses and affect the morphogenetic process to (in time) bring about new morphological traits.

**Table 2** shows an alignment of morphological traits associated with the decrease of multicellularity back toward unicellularity in the SRC upon stress, from a function-based evolutionary perspective. The first type of change in this category is a reduction in photosynthetic surface area. Such changes are induced by the increased stress that occurs along with the increased photosynthetic surface area. Regardless of whether the direct causes of this stress are internal, external, or a combination of these, the resulting changes should be sequential and gradual. Using *Arabidopsis* as an example, sequential changes in organ type are observed, from rosette leaves to cauline leaves to sepals, petals, stamens, and carpels (**Figure 4**). Even among rosette leaves, there are obvious sequential changes in leaf shape and size (Poethig, 1997). In addition, at higher latitudes, day-length and temperature exhibit seasonal changes, imposing additional environmental stress on plants. Traditionally, these changes were separately investigated as phase changes for sequential changes in rosette leaf shape and size (Poethig, 1990), flowering for changes from rosettes to bolting (Bernier et al., 1981; Koornneef et al., 1991), and floral organ identity determination for sequential changes in the four floral organ types (Coen and Meyerowitz, 1991). Such a separation was pragmatically sound in the last century. Therefore, these changes have received tremendous amounts of attention, and great progress has been made in understanding the underlying genetic mechanisms. However, a continuity of organ-type changes has been also noted (Bernier et al., 1981; Lord et al., 1994). Such continuity has been supported at the molecular level, as miR156 play roles in both heterophylly and flowering (reviewed by Poethig, 2013). From the perspective of the SRC, the continuity viewpoint might represent a better description of this process than the traditional ones described above, and it is possible to integrate all of the data generated separately into this new paradigm (Bai, 2016; **Figures 1**, **3**, **4**).

**Table 2:** Alignment of morphological traits associated with the decrease of multicellularity back toward unicellularity in the SRC upon stress from a function-based evolutionary perspective.

	Subcategories	Underlying factors				Morphology observed	Reference
Back to unicellularity in the SRC	Reduction in photosynthetic surface area	Response to internal signals	Physical (mechanic pressure)	Turgor		Size and shapes	Buchanan et al., 2000
				Cell wall
				Gravity
			Chemical	e.g., Sugar, miRNA
		Response to seasonal signals	Photoperiod	Diploid		Sequential changes in organ types (including heterophylly, flowering and floral organ formation in angiosperms) in response to respectively or in combination upon exposure to signals	Buchanan et al., 2000; Hohe et al., 2002
			Temperature
				Haploid		Mainly observed for induction of germ cell/“-ium” differentiation, not reduction in photosynthetic surface area, probably due to lack of observation
			Others

	Induction of germ cells from somatic cells	DGC	Homospory			Bryophytes and some of pteridophytes	Cove, 2005
			Heterospory	Micro-		Some of pteridophytes and spermatophytes	Gifford and Foster, 1989
				Mega-
		HGC	Isogamete			Non-existent in land plants	Scagel, 1984
			Anisogamete	Small	Mobile	Sperm cells in bryophytes and pteridophytes
					Non-M	Sperm cells in spermatophytes
				Large		Eggs

	“-ium” formation: supportive and protective	Diploid: sporangium	Homo	Terminal at axis		Singular or clustered in bryophytes, *Psilotum*, etc.	Scagel, 1984
					Distributed in foliage	Clustered in diverse patterns in ferns	Tanahashi et al., 2005;
			Hetero	Micro-	1-D (dimension)	Singular at terminus, e.g., *Selaginella*	Schulz et al., 2010;
					2-D	Clustered on foliage structures, basal angiosperm stamens	Li et al., 2013
					3-D	Clustered, e.g., eudicot and monocot stamens
				Mega-	Spore-dispersal	Lycophytes
					Spore-retained	Ovules in spermatophytes with various modifications
		Haploid	Antheridium		Elaborated	Bryophytes and pteridophytes
					Reduced	Spermatophytes
			Archegonium		Elaborated	Bryophytes and pteridophytes
					Reduced	Gymnosperms
						Angiosperms


The second type of morphological traits in the decrease of multicellularity category involves the transition from somatic cells to germ cells. These changes essentially occur at the unicellular level and will not be discussed here in detail. However, since all of these changes occur in cell clusters in either diploid or haploid multicellular structures, they are considered here to represent a single subcategory.

The third type of morphological trait in this category involves those I collectively refer to as “-ium” formations: the sporangium, antheridium, and archegonium (for convenience, not exactly taken from their Latin or Greek suffixes). In unicellular eukaryotes, somatic cells are directly induced to undergo meiosis or heterogametogenesis. In multicellular eukaryotes, such transitions/differentiation occur in specific multicellular structures and receive support in nutritional supply and protection against environmental stresses. Therefore, the structures utilized to support and protect cells committed to undergoing meiosis and heterogametogenesis, i.e., the protective “-ium” structures, understandably exhibit new morphological traits consistent with their functions.

The induction of germ cells and reproductive organs after a period vegetative growth is a widely accepted concept in plant biology. However, how such a sequential process evolved is a matter of controversy. A recent finding triggered the idea of the SRC-derived double ring mode to describe plant development: in rice stamens during development, the MADS protein OsMADS58 (annotated as a C-class protein required for stamen and carpel identity determination) binds photosynthetic genes, inhibits their expression, and participates in establishing the hypoxia niche (Chen et al., 2015). This finding, together with the finding that hypoxia triggers meiotic fate determination in maize (Kelliher and Walbot, 2012), suggests that in the first interval of the SRC (from zygote to meiotic cell formation), photosynthesis and DGC induction are mutually exclusive. Sequential changes in organ types may ultimately be determined by the balance of two opposing driving forces: photosynthesis and stress responses.

### Morphological Traits Associated with Sex Differentiation

Sexual reproduction cycle, a mechanism that eukaryotes ultimately use to adapt to environmental changes, comprises three core events: meiosis, fertilization, and heterogametogenesis (Bai and Xu, 2013; Bai, 2015a). The key functions of heterogametogenesis can be thought of as harnessing genetic variations and simultaneously enhancing heterogeneity by labeling meiotically produced haploid cells (Bai, 2015a). From this point of view, so-called “sex differentiation” refers not to the germ line/cells themselves (as they are already progenitor cells for heterogametogenesis) but rather to mechanisms occurring in the soma of multicellular eukaryotes to ensure heterogametogenesis. Such mechanisms fulfill two basic functions: (1) setting divergence points, which determine the differentiation of somatic organs into male or female organs (e.g., antheridia and archegonia in plants and testis and ovaries in animals) and (2) niche establishment, which helps support and protect germ cell differentiation.

In animals, only one set of multicellular structures is interpolated into the first interval (diploid) of the SRC. In the dichotomous mode strategy (**Figures 2**, **3**), cell lineages for germ cells and soma diverge during early embryogenesis. Heterogametogenesis is carried out by the germline after it migrates into the gonads and is determined by the sexual identity of the gonad. Therefore, sex differentiation can be viewed as a mechanism occurring in diploid soma that centers on gonad differentiation.

In plants, by contrast, multicellular structures are interpolated into both intervals of the SRC (**Figure 2**). Therefore, two transitions from somatic cells to germ cells (DGC and HGC) occur in two multicellular structures, sporophytes and gametophytes respectively. The first transition, which occurs in sporophytes, results in the production of meiotic cells, whereas the second transition, which occurs in gametophytes, results in gamete cell production. If we accept the above definition of sex differentiation as mechanisms occurring in the soma to ensure heterogametogenesis, only the differentiation of antheridia and archegonia in gametophytes of bryophytes and pteridophytes, the second interval of the SRC, satisfy both functions that ensure heterogametogenesis (**Table 3**).

**Table 3:** Alignment of morphological traits associated with sex differentiation from a function-based evolutionary perspective.

	Subcategories	Underlying factors	Morphology observed	Reference
Sex differentiation	Real	Heterogametogenesis	An event in SRC and differentiation at the unicellular level	Bai, 2015a
		Structures ensure HG in gametophytes	Hermaphroditic gametophytes: antheridium and archegonium differentiate in the same gametophyte	This paper
		Precocious divergence of An/Ar differentiation prior to initiation of An/Ar during gametophyte development	Dioecious gametophytes: antheridia and archegonia differentiate in separate gametophytes	
	Pseudo	Heterosporangia, as a sporophyte structures, function in setting divergence point for heterogametogenesis	Micro- and mega-sporangia in Lycophytes	This paper
			Microsporophyll/stamens and ovules in spermatophytes	Gifford and Foster, 1989


Stamens and ovules in angiosperms are essentially elaborated heterosporangia. The differentiation of these organs occurs during the first interval of the SRC, between zygote and DGC formation, which ensures the successful transition from somatic cells to DGC for meiosis, not heterogametogenesis. Using our definition, such a differentiation process should not be referred to as sex differentiation, as it does not directly lead to heterogametogenesis. However, as the gametophytes of spermatophytes, especially in angiosperms, have a severely reduced cell number, there are no enough cells for antheridia and archegonia differentiation, one of the two functions involved in sex differentiation, i.e., divergence point setting was canalized to be carried out by heterosporogenesis in spermatophytes. Since heterosporogenesis does not directly lead to heterogametogenesis but did fulfil one of the two functions in sex differentiation, it is reasonable to refer to it as “pseudo-sex-differentiation.” By contrast, the differentiation of antheridia and archegonia can be considered “real sex differentiation.”

In additional to the presence of two germ cells in plants, DGC and HGC, the relationship between germ line/cells and the somatic tissues/organs supporting and protecting the germ line/cells also differ between animals and plants. In animals, as described above, germline and somatic organ formation initiate separately from a spatiotemporal perspective. By contrast, initiation of germ cells and the somatic cells/tissues surrounding them for support and protection during both the diploid and haploid phases in plants are concurrent spatiotemporally. These differences complicate comparisons of sex differentiation in animals versus plants, even though both types of organisms are derived from the SRC. This is an interesting issue that should be further explored.

### Morphological Traits Associated with Sexual Behaviors

In unicellular eukaryotes, gametes are mobilized in water to facilitate their meeting. In multicellular eukaryotes, gametes differentiate in various protective structures, especially female gametes, i.e., eggs. On the other hand, the key advantage of the SRC is that it autonomously increases genetic variation to help the organism adapt to unpredictable environmental changes. Thus, maintaining proper heterogeneity through heterogametogenesis is an essential property of the SRC (Bai, 2015a). To satisfy both the needs for gametes to meet and to maintain heterogeneity, multicellular eukaryotes must evolve mechanisms to facilitate outcrossing, ensuring proper functioning of the SRC.

Most animal individuals are dioecious and have evolved mechanisms that force individuals to actively search for mating partners to complete their SRC. Such mechanisms are generally referred to as “sexual behavior,” including courtship for intersexual individuals and mating competition for intrasexual individuals. By contrast, plants are sessile and cannot move like animals to help complete the SRC. However, specific multicellular structures have evolved to help fulfill these functions. Morphological traits associated with these functions can therefore be referred to as “sexual behavior” in comparison with that of animals.

**Table 4** lists major morphological traits associated with these process. To facilitate the meeting of gametes, two functions are required: spore dispersal and gamete delivery. Endothecia in sporangia have evolved to facilitate the dispersal of spores, including homospores in bryophytes and pteridophytes and microspores in spermatophytes. For gamete delivery, while sperm cells are mainly delivered simply via water during gametophyte development in bryophytes and pteridophytes, very complicated multicellular structures have evolved for sperm delivery in spermatophytes. For example, the pollen tube has evolved as a carrier of sperm, with astonishingly complicated behaviors. Other prominent morphological traits include the structures in angiosperm sporophytes, including the stigmas of carpels for pollen collection and petals and associated structures to attract pollinators. The latter exhibit tremendous, fascinating variations that have evolved during interactions with pollinators. Three mechanisms are used to maintain heterogeneity or to promote outcrossing, i.e., self-incompatibility, the production of morphological differences in reproduction organs, and unisexual flower production. While self-incompatibility mainly results from invisible genetic mechanisms, the other two mechanisms are mainly associated with the specification of morphological characteristics.

**Table 4:** Alignment of morphological traits associated with sexual behaviors from a function-based evolutionary perspective.

	Subcategories	Underlying factors						Morphology observed	Reference
Sexual behavior	Facilitation of gamete meeting	Diploidy	Environmental interactions	Protection of sporangia, for both groups in spermatophytes				Ovulifeous scales and carpels	Gifford and Foster, 1989
				Platform for pollen-collection, only for angiosperms				Stigma in carpels	Scagel, 1984
			Spore-dispersal	In sporangia of homosporous, heterosporous in non-seeds plants and of microsporophyll and stamen in spermatophytes				Endothecium
			Sophisticated structures facilitating gamete meeting	Attraction of pollinators, petals and sepals		Visual	Colors, shapes	Colored spots, etc.
					Olfactory	Volatile materials
						Taste	Secreted components	Nectary
				Interaction of M/F partners			Sperms/egg
							Pollen tube/female parts
							Pollen/stigma
		Haploidy		Sperm mobility in bryophytes, pteridophytes and a few gymnosperms					Gifford and Foster, 1989
				Sperm delivery: pollen tubes (derived from filament structures in moss)			Attraction		Higashiyama and Takeuchi, 2015
							Guidance
							Burst and check

	Promotion of outcrossing	Self-incompatibility		Diploidy			Sporophytic		Bai and Xu, 2013
							Gametophytic
				Haploidy					
		Morphological difference		Sporophytes (stamen/ovule)			Spatial	Distyly	Scagel, 1984
							Temporal	Dichogamy	
				Gametophytes			Dioecy		
		Unisexual flowers (USF)		Flowers	Environmentally induced		Internal, e.g., hormonal		Bai and Xu, 2013
							External, e.g., light/temperature/…		
					Genetically fixed		Specific genes		
							Specific loci	Autosome	
								Heterochromosomes	
					Pseudo-unisexual flower			Male sterility	
				Colonies	Mixed USF in a single colony			Monoecious	
					Separated USF in two colonies			Dioecious	


### Morphological Traits Associated with Particular Stress Responses

Some traits have evolved to help plants cope with particular environmental stresses. These traits include those for adaptation to life on land, such as cutin formation (to prevent rapid water evaporation) and archegonia or embryo sacs plus ovules (to protect eggs and zygotes, an important seed trait). Since plants are sessile organisms, one unique way that plants ensure effective energy usage is to maximize the utilization of synthesized materials while minimizing exposure to environmental stress. Some traits appear to be associated with the latter functions, such as senescence, programmed cell death, and abscission of dead organs. The third subcategory is morphological changes to help the plant adapt to specific or extreme environmental conditions, such as the development of enlarged shoot tips or roots for assimilate storage. The morphological traits associated with these functions are listed in **Table 5**.

**Table 5:** Alignment of morphological traits associated with particular stress responses from a function-based evolutionary perspective.

	Subcategories	Underlying factors		Morphology observed	Reference
Adaptation to environmental stresses	Land habitat	Efficient water usage		Cutin	Scagel, 1984
		Trichome and fibers			Smith et al., 2010
		Protection of SRC core cells	Zygote and embryo	Archegonia in non-seed plants and ovules (seeds) in seed plants
			Spore	Spore walls and pollenin	
			Egg and embryo sac	Endosperm (derived from double fertilization in angiosperms)

	Energy saving	Senescence		Color change, structural degradation, abscission, etc.	
		Cell death
		Abscission

	Extreme conditions	Storage organs		Tubers
				Tuberous roots
				Storage stems
		Other abnormal organs		Thorns
				Tendrils
				Abnormal leaves to capture insects


Some morphological changes that occur in plants upon exposure to biotic stress are not discussed here for two reasons: (1) biotic stress responses are highly complicated and are difficult to summarize concisely, and (2) morphological responses induced by pathogen infection appear to result from a combination of regulatory mechanisms that were originally utilized for abiotic environmental stress or internal stress responses (Zhu et al., 2005; Chen et al., 2007; Wang et al., 2007; Campos et al., 2016; Jin et al., 2016; also see reviews of Huot et al., 2014; Chaiwanon et al., 2016), although particular signaling systems have evolved for pathogen recognition, and a huge variety of secondary metabolic pathways have evolved for plant–pathogen interactions.

## Function-Based Morphology: Plant Morphogenesis 123

Almost all morphological traits mentioned in botanical textbooks and the literature are listed in **Tables 1**–**5** but have been aligned in an unconventional manner. It might take some time to become accustomed to the logic and principles underlying such a new alignment. However, based on the “function-based evolutionary perspective,” it is clear that the morphological traits described to date, regardless of species, can be aligned to the “double-ring” process derived from the SRC. As mentioned above, such an alignment of morphological traits demonstrates that the double-ring process can indeed be treated as a frame of reference equivalent to animal embryogenesis, as it functions as a core process shared by all plant species. To further integrate the concepts of the SRC, the double-ring mode, and morphological traits, I propose a new conceptual framework to better understand the process of plant morphogenesis: Plant Morphogenesis 123. This conceptual framework describes plant morphogenesis on three levels:

Level one is ONE starting point: the SRC. All plants are multicellular eukaryotes, with morphogenesis derived from the SRC, a modified cell cycle representing the ultimate mechanism for environmental adaptation. This concept explains why all plants possess core cells (zygote, meiotic cells, and gametes) and undergo core unicellular events (meiosis, heterogametogenesis, and fertilization). This concept also explains the relationship between multicellular structures and the unicellular SRC: interpolation of multicellular structures into the two intervals between the three core cells (**Figures 1**, **2**).

Level two consists of TWO themes. One theme is the method of building of multicellular structures. Since the L-system is a successful way to describe plant morphogenesis (Prusinkiewicz and Lindenmayer, 1990; Prusinkiewicz and Runions, 2012), there must be corresponding molecular mechanisms underlying this process. The other theme is the regulation or driving forces of changes in morphological structure. As discussed above, most morphological changes (represented by the morphology of lateral organs that initiate from growth tips) are ultimately driven by two forces: photoautotrophy and stress responses.

Level three is the most complicated, representing THREE sequential steps in morphogenesis during the completion of the plant life cycle. The first step is photoautotrophism driving an increase in surface area for photosynthesis and away from the unicellularity of the SRC. The second step is the increased external and internal stress that accompanies the increase in the surface area available for photosynthesis. The third step involves this increase in stress driving a reduction in the surface area available for photosynthesis and compelling the morphogenesis back toward the unicellularity of the SRC.

Through Plant Morphogenesis 123, the life cycle is completed, a PDU forms, and numerous PDUs are integrated into the colony that we refer to as a plant.

Using Plant Morphogenesis 123 as a frame of reference, it becomes obvious that some fundamental issues in plant morphogenesis have not yet been properly addressed. One issue is that little is known about the generally applied molecular mechanisms underlying so-called “axial growth” proposed by Prusinkiewicz and Lindenmayer (1990) (**Table 1**), although such a model has been successfully used for computer simulation of plant morphogenesis and is supported by some molecular data (Prusinkiewicz et al., 2007, 2009; Bayer et al., 2009; Bilsborough et al., 2011; O’Connor et al., 2014; Yoshida et al., 2014; Zadnikova et al., 2016). Since this mechanism is so fundamental for the formation of multicellular structures, many more investigations of this topic are expected.

Another issue is the multicellularization of growth tips. Much effort has been devoted to genetic analysis of the organization of the shoot apical meristem in angiosperms (Barton, 2010; Pautler et al., 2013; Tanaka et al., 2013; Tameshige et al., 2015). However, single or double cells function quite well as growth tips to generate lateral organs in both bryophytes and pteridophytes. How do the growth tips become multicellularized in spermatophytes? While multicellularization is clearly an important evolutionary innovation, little is known about this process, although some efforts have been made to this end (review see Plackett et al., 2015).

Finally, from a more traditional viewpoint, morphogenesis in angiosperms can be divided into two phases, vegetative and reproductive, with flowering representing a transition point. However, according to Plant Morphogenesis 123, all organ types interpolated into the interval between zygotes and meiotic cells, such as in angiosperms, are sequentially generated, with modifications driven by two forces: photoautotrophism and stress responses. Flowering involves only part of this series; photoperiodic responses and vernalization are the main additional mechanisms used by plants growing at higher latitudes to adapt to seasonal changes in the environment. Using this new conceptual framework, I am optimistic that the ultimate regulatory mechanisms underlying morphological changes during the entire (not partial) process will be discovered.

## Conclusion

There is a common saying that “seeing is believing.” This is true in some circumstances. However, our human-centered viewpoint has brought about an inappropriate frame of reference for interpreting plant morphogenesis, i.e., viewing a plant as an individual equivalent to an individual animal. Although tremendous progress has been made in describing and interpreting plant morphogenesis and in deciphering its underlying molecular mechanisms, some fundamental questions remain. Among these are whether a determinate program underlies plant development, whether there is a common process equivalent to animal embryogenesis shared by diverse plant species following the divergence of photoautotrophic organisms from a common ancestor, and what are the key evolutionary innovations underlying the divergence of the major lineages.

Looking back through the history of the study of plant morphology, it is clear that such questions have originated from human-centered observations and interpretations of this process. Therefore, without changing the historical perspective of plant morphology, the puzzle cannot be solved. By echoing and reviving the classic way of observing and interpreting plant morphology proposed by the founding fathers of modern botany, i.e., to view a plant as a colony of developmental units (Waddington, 1966; Bai, 1999), I developed Plant Morphogenesis 123 as a new conceptual framework for plant morphogenesis. Using this framework, morphological traits are aligned following the SRC-derived double ring mode. From this function-based evolutionary perspective, we can better identify the evolutionary significance of any morphological trait in plants. In turn, it becomes easier to identify which morphological traits are important for understanding key evolutionary innovations.

This conceptual framework is undoubtedly unfamiliar to most readers as it, and indeed the concept of the SRC, is so new. According to Gifford and Foster (1989), plant morphology studies have gone from the “casual inspection on surface aspects of plants” to “systematic inspection of hidden aspects of form, structure, and reproduction that constitute the basis for the interpretation of similarities and differences among plants.” From this perspective, the exploration of nature is similar to assembling a jigsaw puzzle: one can carefully examine the picture on the box, diligently collect and examine the pieces, and properly assemble the pieces together according to the picture. In exploring nature, data must be collected diligently and assembled carefully and properly as well. The only difference is that there is no one fixed “picture” used as a reference for data assembly. Therefore, it is not surprising that when a conceptual framework no longer provides a solid basis for assembling or integrating new data, a change in the conceptual framework or paradigm shift should be considered. Using a new conceptual framework, available data can be realigned to obtain a better “picture.” More importantly, if the paradigm shift is appropriate, new opportunities for exploration will emerge.

## Author Contributions

The author confirms being the sole contributor of this work and approved it for publication.

## Conflict of Interest Statement

The author declares that the research was conducted in the absence of any commercial or financial relationships that could be construed as a potential conflict of interest.

## Footnotes

1 http://algorithmicbotany.org
